# An Animal Model of Type A Cystinuria Due to Spontaneous Mutation in 129S2/SvPasCrl Mice

**DOI:** 10.1371/journal.pone.0102700

**Published:** 2014-07-21

**Authors:** Marine Livrozet, Sophie Vandermeersch, Laurent Mesnard, Elizabeth Thioulouse, Jean Jaubert, Jean-Jacques Boffa, Jean-Philippe Haymann, Laurent Baud, Dominique Bazin, Michel Daudon, Emmanuel Letavernier

**Affiliations:** 1 Sorbonne Universités, UPMC Univ Paris 06, UMR S 702, Paris, France; 2 INSERM, UMR S 702, Paris, France; 3 Department of Physiology and Biophysics, Cornell University, Ithaca, New York, United States of America; 4 Biochimie, AP-HP, Hôpital Trousseau, Paris, France; 5 Institut Pasteur, Mouse Functional Genetics Unit, Paris, France; 6 CNRS URA 2578, Paris, France; 7 Néphrologie, AP-HP, Hôpital Tenon, Paris, France; 8 Explorations Fonctionnelles Multidisciplinaires, AP-HP, Hôpital Tenon, Paris, France; 9 CNRS-LCMCP- Sorbonne Universités UPMC Univ Paris 06, Collège de France, Paris, France; UCL Institute of Child Health, United Kingdom

## Abstract

Cystinuria is an autosomal recessive disease caused by the mutation of either *SLC3A1* gene encoding for rBAT (type A cystinuria) or *SLC7A9* gene encoding for b^0,+^AT (type B cystinuria). Here, we evidenced in a commonly used congenic 129S2/SvPasCrl mouse substrain a dramatically high frequency of kidney stones that were similar to those of patients with cystinuria. Most of 129S2/SvPasCrl exhibited pathognomonic cystine crystals in urine and an aminoaciduria profile similar to that of patients with cystinuria. In addition, we observed a heterogeneous inflammatory infiltrate and cystine tubular casts in the kidney of cystinuric mice. As compared to another classical mouse strain, C57BL/6J mice, 129S2/SvPasCrl mice had an increased mortality associated with bilateral obstructive hydronephrosis. In 129S2/SvPasCrl mice, the heavy subunit rBAT of the tetrameric transporter of dibasic amino acids was absent in proximal tubules and we identified a single pathogenic mutation in a highly conserved region of the *Slc3a1* gene. This novel mouse model mimicking human disease would allow us further pathophysiological studies and may be useful to analyse the crystal/tissue interactions in cystinuria.

## Introduction

Urolithiasis is one of the most frequent diseases worldwide and affects more than 10% of the population in developed countries [1], [2]. Cystinuria is an autosomal recessive disorder leading to cystine stone formation in kidneys and accounts for 1–2% of all cases of urolithiasis. Cystinuria holds a special position among renal stone diseases due to a high recurrence rate, associated to a frequent progression toward chronic kidney disease and renal failure [3]. Two different types of cystinuria may be distinguished according to genetic: type A cystinuria, due to mutations along the *SLC3A1* gene encoding the rBAT heavy subunit and type B cystinuria, with mutations in the *SLC7A9* gene encoding for light subunit b^0,+^ AT [4]. The two subunits, rBAT (96 kDa) and b^0,+^AT (40 kDa), are linked together to form the transporter of dibasic amino acids in the brush border of the proximal tubule [5]. In vitro studies have proven that rBAT dimerizes with b^0,+^AT which provides the catalytic activity of the transporter. Subsequently, the dimer acquires a glycosylated form and then its final tetrameric structure. [6], [7].

In both types A and B cystinuria, mutations result in a defect of reabsorption of the dibasic amino acids and cystine [4], [5]. The excessive amount of cystine in urine leads to a high risk of cystine precipitation and to the formation of stones as a consequence of the poor solubility of this aminoacid. Current treatments are based upon restriction of methionine and salt intakes to reduce cystine excretion, diuresis increase, and urine alkalinisation. In addition, sulfhydrils like D-Penicillamine may be useful: they form soluble complexes with cysteine moieties but their use is limited by side-effects [8], [9]. Beyond this preventive medical treatment, patients need frequent urological treatment for relapsing stones and eventually develop chronic kidney disease.

For the first time, we have observed renal stones in a commonly used mouse strain, 129S2/SvPasCrl. Since we observed that 129S2/SvPasCrl stones share similarities with human cystine stones, we characterized 129S2/SvPasCrl mice phenotype and their urolithiasis process.

## Material and Methods

### Mice

Ten 7-weeks old 129S2/SvPasCrl male mice and ten 7-weeks old C57BL/6J male mice were purchased from Charles River Laboratories (Arbresle, France) and housed in Tenon Hospital. Two 129S1/Sv mice were purchased from the Jackson laboratory (USA). Five 129S2/SvPas male mice and five 129S2/SvPasCrl mice were bred and housed in Pasteur Institute (Paris, France). All efforts were performed to reduce animal suffering. They were housed in similar conditions (5 mice/cage) with a 12-h dark/light cycle and fed ad libitum on standard mouse chow. Environmental enrichment was routinely performed. All animal procedures of the laboratories are performed in accordance with the European Union Guidelines for the Care and Use of laboratory animals and with local Institutional Animal Care and Use Committees (“comité d'éthique en experimentation Charles Darwin C2EA-05” and Pasteur Institute ethical committee) guidelines. Since there was no experimental procedure until sacrifice, no specific authorization was required from ministry or local ethical committee according to European convention STE-123 and French legislation (décret 2001-486). Animals were anesthetized with a lethal intraperitoneal pentobarbital injection to minimize suffering.

### Biochemistry

Urines have been collected at 9, 11 and 13 weeks during 18 hours in metabolic cages with free access to water. Urine osmolarity has been measured by an Osmometer 3320 (Advanced instruments). Blood has been collected at the time of the sacrifice. Serum creatinine has been analyzed by enzymatic methods with a Konelab 20 automate (ThermoScientific).

### Crystalluria and aminoaciduria

Between 8 and 12 weeks, fresh urine collected after spontaneous voiding was quantitatively analyzed for crystalluria by a trained technician. The cystine crystal volume was assessed by the following formula: [(mean size (µm) of 20 crystals observed)^2^ * number of crystals in a Malassez cell * 0.65] [10]. Urinary pH was measured at the same time. The day before sacrifice, fresh urine was collected from five 129S2/SvPasCrl and three C57Bl/6J mice to analyze aminoaciduria. Fresh urine from five 129S2/SvPas and five 129S2/SvPasCrl housed in Pasteur Institute mice was collected and analyzed in similar conditions. Urine amino acid concentrations were determined using ion-exchange chromatography followed by colorimetric ninhydrin detection at 570 nm and 440 nm (Jeol Aminotac). Urinary amino acid concentrations were normalized to urine creatinine concentration (Crea Plus Roche Diagnostics with a Modular P automate).

### Fourier transform infrared (FTIR) spectroscopy and scanning electron microscopy (SEM)

Calculi in the bladder were collected during the necropsy, and weighed. They were observed with a SEM (Zeiss Supra 55VP). Calculi composition was analyzed by Fourier transform infrared spectrometry (Bruker optics). Three µm-thick frozen kidney sections were performed and spread on low-e microscope slides (MirrIR, Kevley technologies), and FTIR measurements were carried out at SOLEIL Synchroton (St Aubin-Gif sur Yvette, France). The infrared microscopic mapping was collected in reflection mode, using an infrared microscope (Niplan-Thermo Nicolet) coupled to a FTIR spectrometer (Magma 500-Thermo-Nicolet). The compounds were identified by comparing them to reference spectra [11].

### Immunohistochemistry (IHC) and morphometric evaluation of fibrosis

Kidney tissues were fixed in AFA and formalin and embedded in paraffin. Three µm-thick paraffin sections were rehydrated. After blockade of endogenous peroxydase (Dako), sections were stained with rabbit anti-rBat (Santa Cruz Biotechnology, 1/200), Goat anti-rBat (Santa Cruz biotechnology, 1/800), rabbit anti-CD3 (Dako, 1/200), rat anti-F4/80 (Abd serotec, 1/400) followed by HRP-coupled anti-rabbit, anti-goat or rat-IgG (Nichirei biosciences inc). Staining was revealed by AEC chromogene (Dako) and counterstained by haematoxilin. Kidney fibrosis was assessed by circularly polarized light analysis of picrosirius red stained sections. Anti-F4/80 and picrosirius red stained sections were quantified using computer-based morphometric Analysis software. The percentage of positive area was measured in all cortical fields for five animals from each strain (x200). A mean value of the percentage of positive area was calculated for each kidney.

### Western Blots

Proteins were obtained from the kidney using RIPA buffer according to the manufacturer's instructions. Protein concentration was measured according to the standard Bradford technique. Twenty µg of protein were separated by electrophoresis on NovexBisTris 4–12% gels (Invitrogen), and transferred onto a PVDF membrane (Biorad). The membrane was probed with polyclonal rabbit anti-rBat (Santa Cruz biotechnology, 1/100), rabbit anti-b^0,+^AT (Bioss Inc, 1/1000), rabbit anti-GAPDH (Sigma, 1/160000) or polyclonal rat anti Hsp70 (1/1000), followed by secondary anti-rabbit and anti-rat peroxydase-linked antibody (Amersham, 1/4000) and peroxydase detection.

### Real-Time quantitative PCR (RT-q-PCR)

RT-q-PCR was used to measure the levels of mRNA of *Slc3a1* and S*lc7a9* using *Hprt* as standard in kidneys. Total RNA was extracted using Trireagent (MRC). The concentration and purity of RNA was determined by measuring the absorbance at 260 and 280 nm. DNA was removed by DNase I treatment for 30 min at 37°C (Fermentas). One µg RNA was reverse-transcribed into cDNA (Invitrogen). Amplification was performed with the Light Cycler 480 (Roche) using SYBR green. Reactions were cycled 42 times (denaturation 95°C, annealing 58°C,extension 72°C). The following oligonucleotide primers were used:

*Slc3a1* exon1: F: TGGGAATGGAGACCTGAAAG, R: TGATTTATAAAAGGAAGTGATCCAAA; *Slc3a1* exon3: F: CCATGTCAACGGTGTAACCA, R: GCCAGCTGGAGTTTCCATAC;

*Slc3a1* exon9: F: ACACCGTCAATGTGGATGTC, R: TCTCTCAAGAGGCAAAACCAG;

*Slc7a9*: F: AACGGAGCTCTTGCAGTCC, R: CCCAAGATGCTGGATAGAGAA;

*Hprt*: F: GGAGCGGTAGCACCTCCT; R: CTGGTTCATCATCGCTAATCAC.

Results are expressed as 2^−ΔΔCT^.

### Gene sequencing

Purification of total DNA from kidney was performed with Qiagen Dneasy blood and tissues spin column protocole. Primers were purchased from Eurogentec and we used the Dream Taq DNAse polymerase (Fermentas). PCR amplifications were carried out with 4 µL genomic DNA at 70 ng/µL (3 min at 95°C, followed by 40 cycles of 30 sec at 95°C, 30 sec at 58, 60 or 62°C, 1 min at 72°C). For exon 4, which contains a GC-rich sequence, we added 2% DMSO. We used a 1.5% agarose gel to verify that the PCR products were indeed generated. The sequencing was performed by Beckman Coulter Genomics (UK).

### Rapid single nucleotide polymorphism (SNP)-based genotyping test

Once the mutation has been identified, a SNP-based genotyping test has been developed to distinguish 129S2/SvPas A/A “mutant” and 129S2/SvPas G/G mice bred in Pasteur institute. DNA was extracted from tail biopsies: proteinase K (20 mg/ml, Invitrogen) digestion at 56°C for 2 h, followed by heat inactivation at 95°C for 10 min. Genotyping was performed by pyrosequencing on a Pyroseq PSQ96MA (Biotage/QIAGEN). Pyrosequencing was centered on the SNP [G/A] in position 1232. Primers used were as follow:

Forward-5′- GTTTGCCAACTCTTCCAGGTTC;

Reverse-biotinylated-5′-GCCATAGTACATCATGGTCCTCTC;

Sequencing-5′- CAGGTTCATGGGGGC.

PCR amplifications were carried out with forward and reverse-biotinylated primers using 5 µl genomic DNA (3 min at 95°C, followed by 50 cycles of 15 sec at 95°C, 30 sec at 60°C, 30 sec at 72°C and 5 min at 72°C). Amplified fragments were captured with Streptavidin Sepharose beads (GE HealthCare) and sequenced with sequencing primer using the PyroMark Gold Q96 Reagent Kit (QIAGEN).

### Statistical analyses

Data are expressed as mean +/− SD. Mann-Whitney and Fisher's exact tests were used to compare values between strains. The level of significance was set to <0.05. A log-rank test was performed to analyze survival.

## Results

### 129S2/SvPasCrl mice are affected by cystine urolithiasis

**Figure 1:**
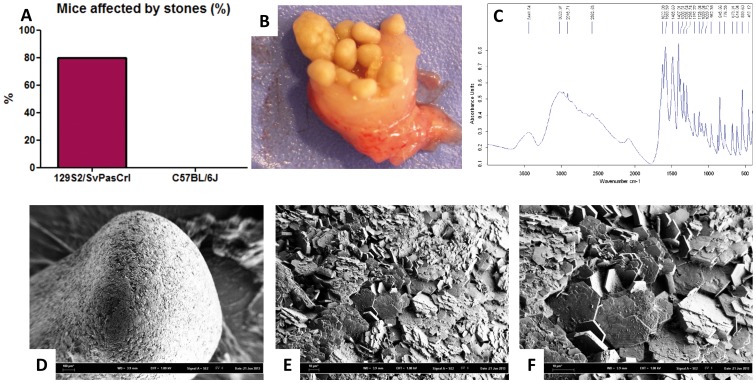
129S2/SvPasCrl mouse is affected by cystine urolithiasis. **A**, Eighty percent of 129S2/SvPasCrl mice sacrificed at 16 weeks or dead before 16 weeks have been affected by urolithiasis whereas no C57BL/6J mice has been affected, p = 0.0007, n = 10/group. **B**, Bladder stones in a 129S2/SvPasCrl mouse. **C**, Spectrum of cystine was obtained by FTIR analysis of mouse stones. **D–F**, Scanning electron microscopy of a stone, at x58, x506, and x1070 magnification respectively, revealed the typical flat hexagonal structure of cystine crystals.

Necropsy revealed stones in 80% of 129S2/SvPasCrl mice but not in any C57BL/6J mouse sacrificed at 16 weeks or dead before (Figure 1A, p = 0.0007). Most of them were observed in the bladder and various degrees of ureteral obstruction have been observed, especially in mice dead before 16 weeks (Figure 1B). The mean weight of stones was 56.5 mg/animal. Scanning electron microscopy was performed in stones from three animals and revealed typical flat hexagonal crystals (Figures 1D–F). The Fourier transform infrared spectrometry (FTIR) confirmed the cystine nature of stones (Figure 1C).

### 129S2/SvPasCrl mice crystalluria and aminoaciduria

**Figure 2:**
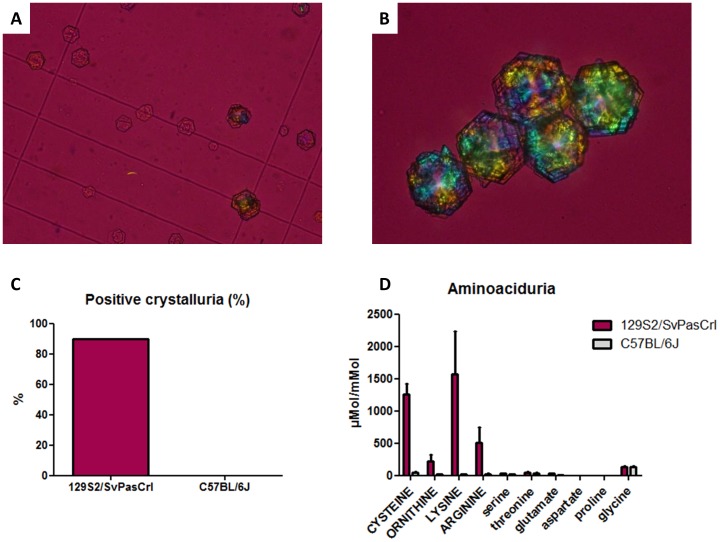
129S2/SvPasCrl crystalluria and aminoaciduria are similar to those of human patients with cystinuria. **A–B**, Typical flat hexagonal cystine crystals were present in 129S2/SvPasCrl fresh urine. **C**, Ninety percent of 129S2/SvPasCrl mice presented crystalluria (p = 0.0001 vs C57BL/6J mice). **D**, Urinary aminoacid chromatography has been performed in 129S2/SvPasCrl mice (n = 5) and C57BL/6J mice (n = 3). Dibasic aminoacids including cysteine (reduced form of cystine) were significantly higher in 129S2/SvPasCrl mice urine (p = 0.036 for each dibasic aminoacid) whereas other aminoacid renal excretion was similar in both strains. Some representative aminoacids are depicted in Figure 1D. Results are expressed as aminoacid concentration (µMol)/creatinine concentration (mMol) in urines.

129S2/SvPasCrl mice and C57BL/6J mice were bred with conventional food excluding methionine intoxication, whose metabolism results in cystine renal excretion. We did not find any difference between both strains regarding urinary pH, diuresis or urinary osmolality, ruling out the hypothesis that 129S2/SvPasCrl mice would have acid or concentrated urines promoting cystine precipitation (data not shown). Despite relatively alkaline urines, crystalluria performed in fresh urines revealed pathognomic flat hexagonal cystine crystals in 90% of 129S2/SvPasCrl mice but not in any C57BL/6J mouse (p = 0.0001, Figures 2A–C). The mean cystine crystal volume was 21971 µm^3^/mm^3^, far above the 3000 µm^3^/mm^3^ threshold risk value associated to stone formation [10]. We also identified a typical urinary profile in five 129S2/SvPasCrl mice displaying increased urinary excretion of cystine and dibasic aminoacids: arginine, lysine and ornithine, mimicking the aminoaciduria in humans with cystinuria (Figure 2D). This result advocates for a potential mutation in dibasic aminoacid transporter in 129S2/SvPasCrl mice.

### Survival study, renal function and kidney injury

**Figure 3:**
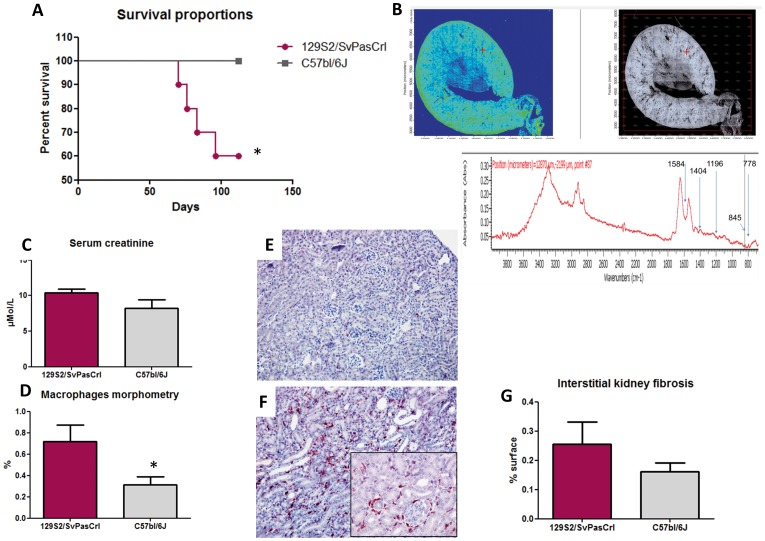
Survival study and kidney injury in 129S2/SvPasCrl and C57BL/6J mice. **A**, Survival proportions of mice from both strains before sacrifice at 16 weeks. 129S2/SvPasCrl mortality was significantly increased (p = 0.045) and necropsy revealed kidney dilation due to urolithiasis. **B**, Infrared cartography of 129S2/SvPasCrl kidney slices revealed the presence of focal cystine aggregates in renal tissues (cystine tubular casts). **C**, Renal function as assessed by enzymatic serum creatinine dosage was not significantly impaired in 129S1/SvPasCrl mice (n = 6) compared to C57BL/6J mice (n = 9) at 16 weeks (p = 0.11). **D–F**, Macrophage infiltrate in kidney tissues was assessed by morphometric analyses of F4-80 immunostaining and was increased in 129S2/SvPasCrl mice in comparison with C57BL/6J (n = 5/group, p = 0.046). Figures 3E and 3F are representative of macrophage infiltrates in C57BL/6 and 129S1/SvPasCrl mice respectively (magnification x200+ zoom). **G**, Fibrosis assessed by sirius red morphometric analysis did not evidence significant fibrosis amount in both strains (percentage of fibrotic area, p = NS).

We analyzed spontaneous survival in 129S2/SvPasCrl and C57BL/6J mice between 8 and 16 weeks. Mortality was significantly higher in the 129S2/SvPasCrl strain (p = 0.045, Figure 3A). The four mice that died before the end of the protocole had bilateral renal dilation and large amounts of bladder stones as observed during the necropsy whereas none of the 16 sacrificed mice had bilateral hydronephrosis, evidencing that death was correlated to bilateral renal obstruction (p = 0.0002). Although we did not observe extensive tubular dilation at 16 weeks in kidneys from both strains, there was evidence for tubular casts in frozen kidney sections from 129S2/SvPasCrl mice. FTIR analysis was performed in these sections by using an infrared analyser, revealing the cystine nature of these casts (Figure 3B). Among the six 129S2/SvPasCrl mice that survived until 16 weeks, we did not observe a significantly higher serum creatinine level in comparison with nine C57BL/6J mice (p = 0.11, Figure 3C), despite an important amount of stones in bladder. Of notice, none of these mice was affected by hydronephrosis. Hematoxylin Eosin staining revealed focally increased cellularity in kidney cortex interstitium of 129S2/SvPasCrl mice due to inflammatory cell infiltrates, including lymphocytes and especially macrophages (Figure 3D–F). However, we did not identify interstitial fibrosis in both strains (Figure 3G).

### Defect of rBAT in 129S2/SvPasCrl mice

**Figure 4:**
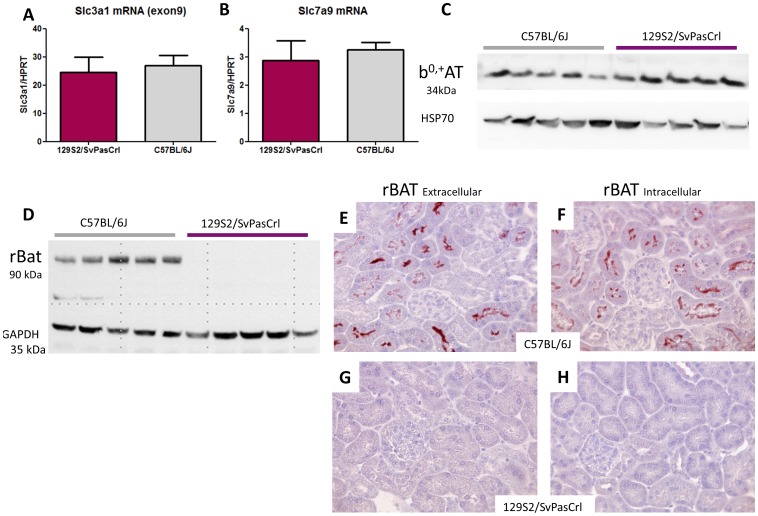
mRNA and protein expression of cystine transporters *Slc3a1*/rBAT and *Slc7a9*/b^0,+^AT in kidney cortex from 129S2/SvPasCrl and C57bBL/6J mice. **A–B**, Quantitative PCR: *Slc3a1* and *Slc7a9* mRNA expression was similar in both strains. **C**, Western Blot: b^0,+^AT was expressed at a similar level in kidney cortex from both strains. **D**, Western Blot: rBAT was expressed in C57BL/6 mice but not in 129S2/SvPasCrl mice. **E–H**, Antibodies directed against the extracellular part of rBAT (Figures 4E and 4G) or against its intracellular part (Figures 4F and 4H) revealed the presence of rBAT at the brush border of proximal tubular cells in C57BL/6J mice (4E and 4F) but not in 129S2/SvPasCrl mice (4G and 4H).

Quantitative PCR revealed a similar *Slc3a1* (tested for 3 exons) and *Slc7a9* mRNA levels in both strains (Figure 4A–B). Western-Blot analysis revealed the presence of b^0,+^AT in the kidney cortex of both strains, whereas rBAT was absent in129S2/SvPasCrl mice (Figure 4C–D). We used two different antibodies against the N- and C-terminal parts of rBAT, revealing by immunohistochemistry the presence of the transporter in the proximal tubule brush border in C57BL/6J mice but not in 129S2/SvPasCrl mice (Figures 4E–H).

### Mutation of *slc3a1* in 129S2/SvPasCrl mice

**Figure 5:**
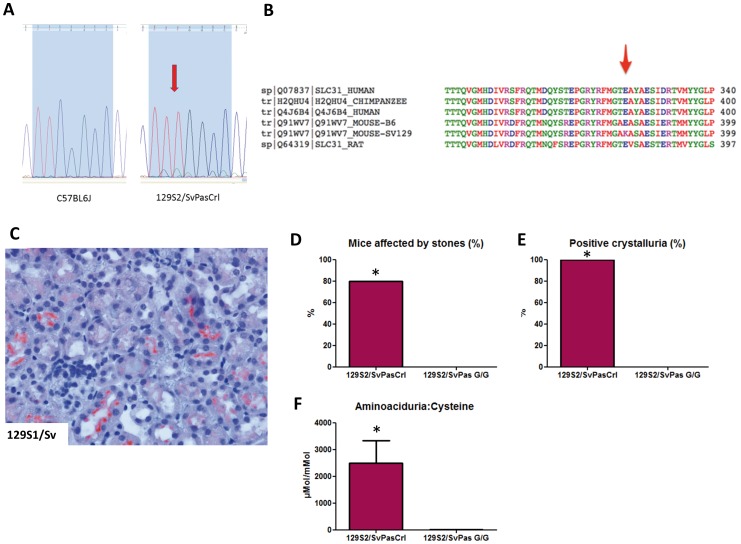
Identification of a functional missense mutation in a highly conserved sequence of the *Slc3a1* gene in 129S2/SvPasCrl mouse. **A**, Mutation of G by A nucleotide at position 1232 in 129S2/SvPasCrl mouse (c.1232G>A). **B**, In position 383, the glutamine is substituted by a lysine in the extracellular part of rBAT protein in 129S2/SvPasCrl mouse. The sequence is highly conserved among mammal species. **C**, 129S1/Sv mice from the Jackson laboratory express rBAT at the brush border of proximal tubular cells. **D**, Eighty percent of 129S2/SvPasCrl mice sacrificed at 10 weeks were affected by urolithiasis whereas no 129S2/SvPas (G/G genotype) mouse was affected (n = 5/group, p = 0.047). **E**, One hundred percent of 129S2/SvPasCrl mice presented cystine crystals in urine whereas no 129S2/SvPas (G/G genotype) mouse was affected (n = 5/group, p = 0.008). **F**, Urinary aminoacid chromatography has been performed in 129S2/SvPasCrl mice and 129S2/SvPas mice (n = 5/group). Cysteine (reduced form of cystine) was significantly higher in 129S2/SvPasCrl mice urine (p = 0.008). Results are expressed as aminoacid concentration (µMol)/creatinine concentration (mMol) in urines.

Sequence analysis of *Slc3a1* genomic DNA from 129S2/SvPasCrl and C57BL/6J revealed a homozygous mutation in exon 7 in 129S2/SvPasCrl mice. The point A1232G mutation is a missense mutation (c.1232G>A) in a highly conserved sequence (Figure 5A). As a consequence, the glutamine in position 383 would be substituted for a lysine (p. E383K, Figure 5B). This substitution stands in the extracellular part of rBAT.

### Functional relevance of the mutation

The glutamine in position 383 is highly conserved among various species, arguing for its functional importance with a SIFT score at 0.03 (Figure 5B). Sequence analysis of *slc3a1* genomic DNA from 129S1/Sv mouse did not reveal any rBAT mutation and the mouse phenome database does not report any specific kidney phenotype in 129S1/Sv mouse (http://phenome.jax.org/db/q?rtn=meas/catlister&req=Ckidney&reqstrainid=3). Overall the nucleotide sequence of the *Slc3a1* gene was similar in 129S1/Sv and 129S2/SvPasCrl mice with the exception of the mutation at position 1232. Immunostainings revealed the presence of rBat protein at the apical brush border in 129S1/Sv mouse purchased from Jackson Laboratory (USA) (Figure 5C). Moreover, we did not detect any cystine crystal in 129S1/Sv mice urine.

To establish definitively that cystinuria was not influenced by genetic background, five 129S2/SvPasCrl “mutant” mice (A/A genotype) and five 129S2/SvPas mice unaffected by the mutation (G/G genotype) were bred and housed in similar conditions and sacrificed at 10 weeks. We did not detect any stone, cystine crystal or significant cystinuria in 10-week old 129S2/SvPas mice expressing rBat whereas 129S2/SvPasCrl mice where all affected by cystinuria and four of them were affected by stones (Figures 5D–F). Few macrophages were evidenced in kidneys from both groups at 10 weeks (not shown).

Accordingly, the mutation is functional and responsible for the loss of rBAT expression and cystinuria in129S2/SvPasCrl mice.

## Discussion

The 129 steel substrains originate from an outcross to C#HeB/FeJ mice carrying the steel-J allele at the mast cell growth factor locus, followed by 12 to 14 generations of backcrossing to 129/Sv [12]. The steel substrain was then distributed to JL Guénet forty years ago (129/SvPas), R Jaenish in 1974 (129SvJae) and M Evans in 1969 (129SvEv). The French substrain was renamed as 129S2/SvPasCrl when Charles River France acquired it in 1996 [12]. Of notice, 129S2/SvPas were maintained segregating the steel-J allele and not exposed to outcrossings or accidental breeding contaminations: their genetic background should theoretically be identical to 129S1/Sv mouse background from the Jackson Laboratory. The 129/Sv mouse substrains were the foundation for establishing ES cell cultures, allowing gene targeting in mice, and became therefore very popular during the past decades since their ES-derived cells can be maintained in a pluripotent state. Simpson et al. highlighted in 1997 the high degree of genetic variations within the 129 substrains and the ES cells derived from them and warned scientific community about the intrastrain genetic variability [12]. Recently, Coulombe et al. reported a spontaneous *Cdt1* mutation in 129 mouse strains (including 129/Ola, 129/J and 129/Sv) responsible for enhanced licensing activity, dysregulating cell-cycle and potentiating *Cdt1* oncogenic properties [13]. On the one hand, the genetic variability among the 129 substrains may be of interest to select a specific background or identify modifier loci but, on the other hand, the potential genetic drift among these substrains may influence experimental models [12]. Models of chronic or acute kidney diseases revealed the importance of 129/Sv genetic background in determining the susceptibility to kidney injury. Indeed, 129/Sv mice are particularly prone to glomerulosclerosis, proteinuria, renal inflammation, and hypertension [14]–[16].

We were initially intrigued to observe a high incidence of urolithiasis in 3- or 4-months old 129S2/SvPasCrl mice. Since urolithiasis may by promoted by either environmental parameters or genetic background, we first compared 129S2/SvPasCrl mice to another classical mouse strain, C57BL/6J mice purchased from the same laboratory, bred and housed in similar conditions. Our observations evidenced that urolithiasis was not linked to environmental parameters. We then demonstrated that such stones result from cystine crystallisation due to high amounts of cystine in urine. The defect of rBAT subunit expression is responsible for the aminoaciduria and we identified a single c.1232G>A mutation in the *Slc3a1* gene responsible for substituting a glutamine for a lysine in position 383, i.e. in the extracellular part of the transporter. At last, the observation that 129S2/SvPasCrl mice were affected by cystinuria, unlike 129S2/SvPas (G/G genotype) mice sharing the same genetic background rules out any doubt about the pathogenicity of the mutation. The 129S2/SvPasCrl substrain may therefore be proposed as a new model for cystinuria type A. We did not find in the European Nucleotide Archive or in the Jackson Laboratory whole genome studies any *Slc3a1* gene mutation in 129/SvJae or 129S1/Sv mice. In addition, we confirmed that 129S1/Sv mice from the Jackson laboratory have a normal expression of rBAT. This is in agreement with the selection of a mutation appearing only in the French substrain and not in all the “steel” substrains. It seems likely that further inbreeding allowed the selection of the mutated recessive allele, increasing progressively the number of cystinuric animals (homozygous for the mutation) over the time.

There are few models of cystinuric mice. Feliubadalo et al. generated a *Slc7a9* knockout mouse resulting in calculi in 42% of the Slc7a9 -/- animals [17]. Ercolani et al. generated a *Slc3a1* knockout mouse of mixed strain background (129/Sv and C57BL/6) and observed stones in all male but not in female knockout animals [18]. Interestingly, we observed cystine crystals in both males and females (not shown) but urolithiasis affected mainly males, a common setting in urolithiasis murine models probably due to anatomical predisposition [19]. To our knowledge, only one mouse model of cystinuria due to *Slc3a1* mutation has been obtained in the past, in an N-ethyl-N-nitrosurea mutagenesis screen for recessive mutations [20]. These *Slc3a1^pbl^* mutants have a mutation leading to an aminoacid exchange D140G in the extracellular domain of the protein. However, rBAT expression has not been analyzed in this model. We also found a homozygous missense mutation causing an aminoacid exchange in the extracellular part of the protein. Although this mutation has not been described in human, it is striking that functional mutations found in type A cystinuria patients are also located in the extracellular domain of rBAT. We observed a normal mRNA expression of *Slc3a1* gene in 129S2/SvPasCrl and C57BL/6J mice, evidencing that the mutation does not affect the transcript levels. rBat protein has not been crystallized and the mutation is responsible for aminoacid substitution in an alpha-amylase like domain, a structure shared by many proteins involved in various tasks, we are therefore unable to predict the impact of the mutation on protein conformation. Bartoccioni et al. have shown in vitro that mutants of the extracellular domain efficiently assemble with b^0,+^AT but fail to acquire the proper tetrameric form [7]. We may therefore hypothesize that rBat mutation is responsible for impaired quaternary structure and subsequently transporter degradation. Of notice, the normal expression of b^0,+^AT at proximal tubular cells brush border in 129S2/SvPasCrl mouse is a proof of concept that rBat does not act only as a chaperone yet is necessary to cystine transport. Overall, we identified a novel cystinuria model whose main interest is to be readily available, allowing testing of new drugs against cystine stone formation. Interestingly, this model is based upon a single missense mutation and differs from knockout murine models. The observation that *Slc3a1* transcripts are normally expressed contrasts with the absence of rBat protein at the brush border of proximal tubular cells, suggesting that a misfolding of rBat protein and/or an impaired organization with b^0,+^AT result in protein degradation as described in vitro. This mouse model would therefore allow to test drugs modifying chaperone expression and potentially allowing rBat re-expression in a murine model mimicking human disease.

Another major focus of interest is the observation that cystine casts are present in tubules and associated with inflammatory infiltrates in 129S2/SvPasCrl kidneys at 16 weeks. The irregular distribution of immune cells in renal interstitium and their association with focally dilated tubules advocates for cystine casts promoting focal lesions upstream of tubular obstruction. One of the specific features of cystinuria, in comparison with other urolithiasis diseases, is the frequent evolution toward chronic kidney disease [3], [21]. Recent reports have evidenced that calcium oxalate crystals precipitation in renal tubules may induce renal inflammation through NLRP3-mediated IL-1β secretion and at term progressive renal failure [22], [23]. Given our results, one may hypothesize that impaired renal function in patients with tubular cystine casts results from inflammatory and fibrotic processes in addition to ureteral obstruction. The 129S2/SvPasCrl cystinuria model deserves further investigations to analyze the crystal/epithelium and matrix interactions in this model.

In conclusion, we describe a novel model of cystinuria, with several strenghts. First these animals are not a knockout model and the single missense mutation in the extracellular part of the protein is similar to pathogenic mutations observed in patients. The normal expression of *Slc3a1* mRNA suggests that quaternary organization of the dibasic aminoacid transporter is impaired. This model also evidences that rBat is not only a chaperone but is necessary for the transporting function of the dibasic aminoacids transporter since b^0,+^AT is normally expressed at proximal tubular cells brush border. Second, these animals are easily available and have a stable genetic background: in a way, the progressive selection of the recessive mutant allele due to inbreeding is a good proof that the strain is really congenic. Third, the proportion of lithiasic animals is very high. Fourth, aminoaciduria, crystals and stones are similar to those of patients, a not so frequent setting in murine urolithiasis models, allowing for instance to test therapeutic drugs. Last but not least, we identified focal tubular cystine casts by FTIR analysis and related focal dilations and inflammatory infiltrates, which could reveal a novel mechanism for progression toward chronic kidney disease in patients with cystinuria and explain why these patients are so prone to renal failure.
